# A Case of Asymptomatic Essential Thrombocythemia in a Child

**DOI:** 10.7759/cureus.28455

**Published:** 2022-08-26

**Authors:** Mohammed Aljuaid, Ziad Alahmadi, Badriah Alasmari, Arwa Alyamani, Eman Khan

**Affiliations:** 1 Paediatrics, Royal Commission Hospital, Yanbu, SAU; 2 Internal Medicine, Umm Al-Qura University, Makkah, SAU; 3 Pediatrics, Armed Forces Hospital, Khamis Mushayt, SAU; 4 Pediatric Hematology and Oncology, King Abdulaziz Medical City, Jeddah, SAU; 5 Hematopathology, King Abdulaziz Medical City, Jeddah, SAU

**Keywords:** reactive thrombocythemia, myelodysplastic syndromes, mpl gene mutation, jak2 negative essential thrombocythemia, essential thrombocythemia

## Abstract

Various factors can be linked to an increase in platelet count. Yet thrombocytosis could be essential. Many genetic mutations have been associated with essential thrombocytosis, which also increases the possibility of myelofibrotic transformation. In pediatrics, essential thrombocytosis is not well-studied. In this article, we present a rare case of a 42-month-old male patient who presented with essential thrombocytosis associated with myeloproliferative leukemia (MPL) gene mutation.

## Introduction

Thrombocytosis is defined as platelets more than 450000/mm3 or greater than two standard deviations of the mean [1]. Different situations can be related to reactive thrombocytosis, including inflammatory processes and infections; in most circumstances, this elevation can be a transient and benign finding [2]. Essential thrombocythemia (ET) is identified If thrombocytosis was sustained in the presence of hyperplasia of megakaryocytes in the bone marrow and was associated with pathogenic mutation after excluding causes of reactive thrombocytosis [3]. In pediatric patients, genetic testing may support the diagnosis of ET, yet it doesn’t rule out primary thrombocytosis in the absence of such mutations. Identified genetic mutations include MPL mutations, JAK2V617F mutation, and PRV-1 expression [4]. Increased risk of myelofibrotic transformation was significantly appreciated with the expression of myeloproliferative leukemia (MPL) gene mutation. Essential thrombocythemia can mimic familial thrombocythemia (FT), but the difference is that FT has markedly lower platelet count and is associated with a lower rate of hepatomegaly and thrombotic events.

## Case presentation

A 42-month-old, previously healthy boy was referred from the primary health care center due to an incidental finding of thrombocytosis while being investigated for chronic abdominal pain. The platelet count was 888,000/mm3 upon referral. The condition was not associated with fever, vomiting, diarrhea, constipation, or weight loss; there was no history of rash, hemorrhage, thrombosis, lymphadenopathy, trauma or surgery, no joint pain, swelling, or difficulty in walking, no changes in the color of urine or amount and no dysuria. No cyanosis, chest pain, or cough, and further systematic review was unremarkable. He had no significant past medical history apart from his functional abdominal pain, which was mild, periumbilical, colicky, and not affecting his daily activity. No significant medication history was reported. His family history was evident for an aunt from his parental side who had leukemia and was treated by bone marrow transplantation (BMT), and the donor was the patient's father. The patient mother claimed to have high platelets but was never investigated. The patient has a family history of three third-degree relatives with sickle cell anemia. Physical examination was unremarkable, aside from mild splenomegaly.

**Figure 1:**
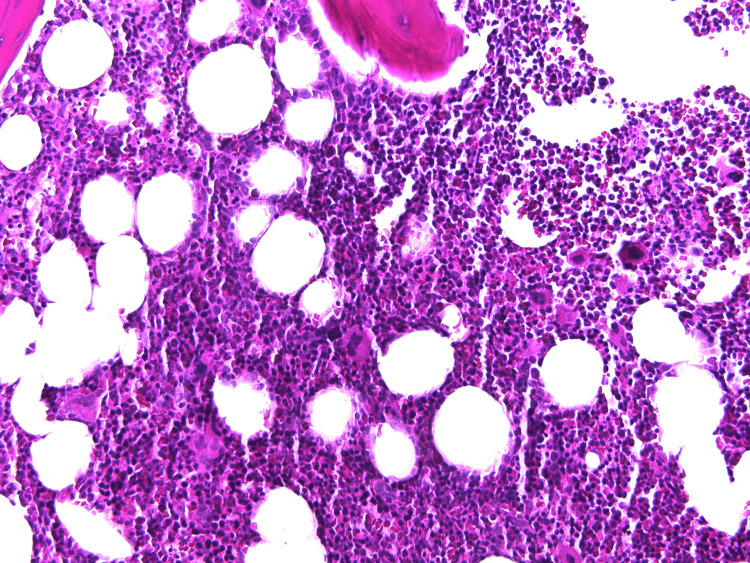
Bone marrow trephine biopsy reveals normocellular bone marrow with increased megakaryocyte proliferation.

**Figure 2:**
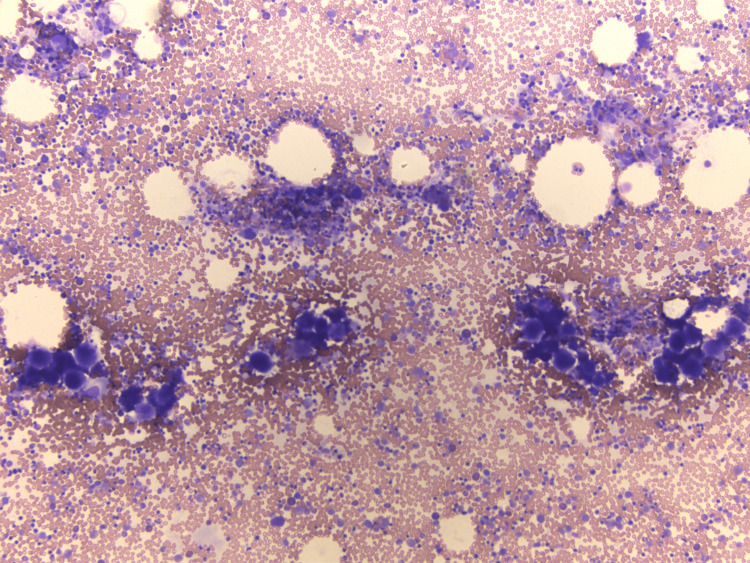
Bone marrow aspirate shows prominent megakaryopoiesis and megakaryocyte clustering.

Investigation showed normal white blood cell count with differential, platelet count of 993000/mm3, erythrocyte sedimentation rate (ESR), C-reactive protein (CRP), and iron profile were within normal ranges, and a sickle screening test was negative. The blood film showed marked thrombocytosis, megakaryocytes, and reactive lymphocytes. A repeated platelet count after one month of the presentation was 1039000/mm3, and after three months was 923000/mm3. Upon screening of other family members, it was found that two of his male siblings had high platelets; their ages were 9 years and 19 years. Bone marrow aspiration of the patient showed increased megakaryocytes, normal cellularity with active erythropoiesis, active granulopoiesis with orderly maturation, and a normal range of lymphopoiesis (Figures 1, 2).

Flow cytometry of the bone marrow sample showed no immunophenotypic evidence of abnormal or aberrant phenotype, and cytogenetics** **of the sample result revealed a normal male chromosome. Genetic testing was positive for MPL gene mutation and negative for Janus Kinase 2 gene (JAK2) mutation.

Outcome

A diagnosis of essential thrombocythemia was made and the family was informed about the translocation to myelodysplastic syndrome (MDS) and counseled about the outcome and their need for follow-up.

## Discussion

As an acute phase reactant, platelets can be elevated with the upregulation of the thrombopoietin (TPO) receptor and interleukin-6 (IL-6) [5]. Thus, reactive thrombocytosis can be relatively common in children. Reactive thrombocytosis could also be secondary to iron deficiency anemia, trauma, or surgery [3]. Affecting 1/1,000,000 child ET is considered rare [6]. Mutations in JAK2V617F or proliferation pathway genes such as JAK-STAT and TPO can lead to ET, which is a myeloproliferative neoplasm (MPN) [6]. Being linked to ET, the myeloproliferative leukemia gene (MPL) participates in the growth and division of megakaryocytes, which is the precursor for platelets via participating in thrombopoietin receptor protein production [6]. In adult patients, mutations of the MPL gene that can lead to ET and MPNs are well described with a risk-stratification system that predicts the risk of progression to acute myeloid leukemia or myelodysplastic syndrome. It also predicts the risk of venous or arterial thrombosis with a stratified management approach and interventions [6]. In contrast to adults, the clinical implications of MPL gene mutations are still not known in pediatric patients.

## Conclusions

Essential thrombocytosis is rare in pediatric population but can be linked to devastating outcomes. This case would raise the question of whether to start aspirin or not in the pediatric population while there is a risk of developing Reye’s syndrome with aspirin in less than 16 years of age. Further case reports and series need to Be identified for prognostication and risk assessment, especially in the pediatric population, as it will help serve to family counseling and developing screening programs. Also, more studies are needed to establish the safety of treatment options for ET and to follow Up for myelofibrotic transformation.
